# Factors Influencing Antibiotic Consumption in Adult Population of Kazakhstan

**DOI:** 10.3390/antibiotics12030560

**Published:** 2023-03-11

**Authors:** Nazym Iskakova, Zaituna Khismetova, Dana Suleymenova, Zhanat Kozhekenova, Zaituna Khamidullina, Umutzhan Samarova, Natalya Glushkova, Yuliya Semenova

**Affiliations:** 1 Department of Public Health, Semey Medical University, Semey 070000, Kazakhstan; 2 Department of Public Health, Asfendiyarov Kazakh National Medical University, Almaty 050000, Kazakhstan; 3 Department of Obstetrics and Gynecology #1, NpJSC “Astana Medical University”, Astana 010000, Kazakhstan; 4 Department of Epidemiology, Biostatistics and Evidence-Based Medicine, Al-Farabi Kazakh National University, Almaty 050010, Kazakhstan; 5 School of Medicine, Nazarbayev University, Astana 010000, Kazakhstan

**Keywords:** antibiotics, antibiotic resistance, awareness

## Abstract

Poor or suboptimal knowledge of appropriate antibiotic use is a cause for global concern and little is known about Central Asian countries. Therefore, this survey is aimed at evaluating awareness about antibiotic use and resistance among the adult population of Kazakhstan. A cross-sectional study of a random sample was conducted between October 2021 and February 2022 among 727 individuals without medical education and followed the methodology described in the WHO report “Antibiotic Resistance: Multi-country public awareness survey”. Half of the respondents (50.4%) received antibiotic therapy within the last 12 months, 40.1% had no prescription for this and 40.4% received no advice from a medical professional. Nearly two-thirds of respondents (65.3%) never heard about antibiotic resistance and 57.2% believed that it is worth requesting the same antibiotic if it helped to treat a similar condition previously. In general, knowledge about antibiotic use proved to be low in 82.1% of respondents and 91.9% agreed with the statement that a common cold requires antibiotics. There is a need for awareness-raising campaigns to improve the knowledge about antibiotic use and resistance in the population of Kazakhstan.

## 1. Introduction

Since the discovery of the first antibiotic (penicillin) by Alexander Fleming in 1928, antibiotics have revolutionized modern medicine by making previously incurable infections and conditions, including pneumonia and other life-threatening bacterial infections, treatable. Today, many routine medical procedures, such as cesarean section, appendix removal, and chemotherapy, rely on effective antibiotics to prevent common infections from becoming fatal. However, decades of misuse of antibiotics and abuse by doctors and patients (to treat mild ailments) and farmers (to promote growth in agriculture and aquaculture) have led to the emergence of antimicrobial/antibiotic resistance (AMR or ABR), which seriously threatens the health of humans, animals, and the environment [1]. In recent years, numerous awareness-raising activities have been undertaken to educate both the public and medical professionals on the issue of unjustified antibiotic consumption, which remains a major public health concern.

Despite a slight reduction in the consumption of antibiotics for systemic use, the irrational use of these drugs remains prevalent in Kazakhstan. This is due to the over-the-counter availability of 27.5% of antibiotics and excessive prescription by medical professionals, where 29.9% of all medications prescribed are antibiotics [2]. The World Health Organization (WHO) recommends a decrease in global antibiotic prescriptions by 20% to combat the development of antibiotic resistance [1]. Additionally, the actual consumption of antibiotics in Kazakhstan is thought to be higher, given the widespread empirical use during the COVID-19 pandemic [3].

The excessive prescription of antibiotics can have significant financial implications for individuals and healthcare systems. When antibiotics are over-prescribed, not only can they become less effective due to the development of antibiotic resistance, but they can also cause side effects that lead to further medical expenses. As antibiotic resistance grows, the cost of treating infections increases, as more expensive and effective drugs are required. This can put a strain on health insurance providers and government healthcare programs, which may struggle to cover the cost of these treatments. The bulk of healthcare provided to the population of Kazakhstan is reimbursed from the state-owned social health insurance fund. Out-of-pocket payments contribute to over-the-counter sales and unnecessary prescriptions may affect the financial stability of the poorest population stratum [1].

Being concerned about the growing trend of antibiotic consumption, the WHO developed a global action plan to combat antibiotic resistance which urges all countries to increase public knowledge about antibiotics and antibiotic resistance through effective information and communication campaigns [4]. Thus, in order to develop effective intervention strategies, it is important to understand the level of awareness, attitudes, and perceptions of the population about antibiotics and antibiotic resistance [5]. We therefore conducted a cross-sectional study to evaluate awareness about antibiotic use and resistance among the adult population of Kazakhstan. In our study, we examined the following hypotheses: (i) there may be age and gender-based differences in awareness about antibiotic use; (ii) rural and urban residents may have different attitudes towards antibiotic use; and (iii) education and income levels may influence the pattern of antibiotic use.

## 2. Results

Table 1 presents an overview of the population under study, detailing their key characteristics. Out of the total sample, 542 individuals (74.6%) identified as female, while 185 (25.4%) identified as male. The majority of participants were under 25 years of age (60.2%) and resided in urban areas (53.6%). Over half of respondents held undergraduate or postgraduate degrees (55.5%). The most common household type was comprised of multiple adults aged > 16 years and at least one child under 16 (40.4% of all households). The median household income was 150,000 Tenge, which is equivalent to 350 US dollars [6].

Table 2 illustrates the level of awareness regarding antibiotic use and resistance by gender. Notably, there was a significant difference in the duration since respondents last received antibiotics between male and female participants. Specifically, 33.1% of females reported receiving antibiotics within the last month, compared to 28.8% of males who reported receiving antibiotics within the last 6 months. Moreover, 55.0% of males believed that they should stop taking antibiotics once they have taken all the prescribed medicine, in contrast to 70.5% of females. Regarding prescription patterns, the majority of females (64.1%) reported having a prescription for the antibiotics they last took, while the majority of males (52.1%) did not.

Furthermore, a greater proportion of females (83.2%) disagreed with the notion that it is advisable to use the same antibiotics as a friend or family member who previously treated similar symptoms or disease, compared to males (65.7%). It is noteworthy that while the vast majority of both males and females obtain antibiotics from a medical store or pharmacy, none of the females and 1.8% of males stored antibiotics from a previous time. Lastly, there was a significant difference between genders in seeking advice from healthcare professionals regarding antibiotic use. Specifically, 63.7% of females sought advice from healthcare professionals, while only 46.8% of males did so.

Between-group comparisons of individuals aged 24 years and younger and their older counterparts (Table 3) are of interest. Individuals aged 24 years and younger were more likely to disagree with the statement that it is good to use the same antibiotic if a friend or family member used it to treat the same symptoms or disease before than individuals 25 years and older (81.9% vs. 73.9%). In our study, younger participants reported hearing about antibiotic resistance significantly more often (68.7%) than older participants did (60.2%). However, individuals aged 25 years and older were more likely to seek advice from a doctor, nurse, or pharmacist on how to take antibiotics than their younger counterparts (65.7% vs. 55.6%). In addition, a significant difference was observed in the time of the last antibiotic intake, with most younger individuals (28.3%) receiving antibiotics in the last month. In contrast, the majority of older individuals (32.2%) received antibiotics in the past 6 months.

According to Table 4, individuals with tertiary education had a prescription for the antibiotics they consumed significantly more often than those with pre-tertiary education (64.6% vs. 53.8%). Most individuals with pre-tertiary education had consumed antibiotics in the past month (30.3%), while the majority of those with tertiary education had taken antibiotics in the past six months (28.9%), which was significant (*p* = 0.039). Individuals with higher education heard about antibiotic resistance insignificantly more often than those with lower education (37.5% vs. 31.2%).

Table 5 presents the between-group comparisons based on the place of residence. The only significant difference was observed in the time of the last intake of antibiotics. Specifically, the majority of rural respondents (36.9%) took antibiotics in the past month, while only a quarter (25.6%) of urban and suburban residents did so. Nearly equal proportions of rural and urban/suburban residents consumed antibiotics within the past 6 months (25.2% vs. 25.9%).

The between-group comparisons based on income level are presented in Table 6. Respondents with income below the median (≤150,000 Tenge) believed significantly less often than their wealthier counterparts that it is advisable to request the same antibiotic if it had previously helped to treat the same symptoms or disease (53.1% vs. 62.6%). Furthermore, they had a lower level of awareness about antibiotic resistance, with only 30.4% of them having ever heard the term “antibiotic resistance”, compared to 40.3% of individuals with income above the study median. Additionally, there were significant differences in the time since the last intake of antibiotics, as the majority of individuals with lower income had consumed antibiotics in the past month, while the majority of individuals with higher income had consumed antibiotics in the past six months.

In general, the level of knowledge regarding antibiotic use was found to be inadequate. A total of 12 questions were asked, and respondents’ knowledge was considered low if they answered six or fewer questions correctly, and good if they answered seven or more correctly. The vast majority of study participants (82.1%) had low knowledge about health problems that can be treated with antibiotics, with only 17.9% answering seven or more questions correctly.

**Figure 1:**
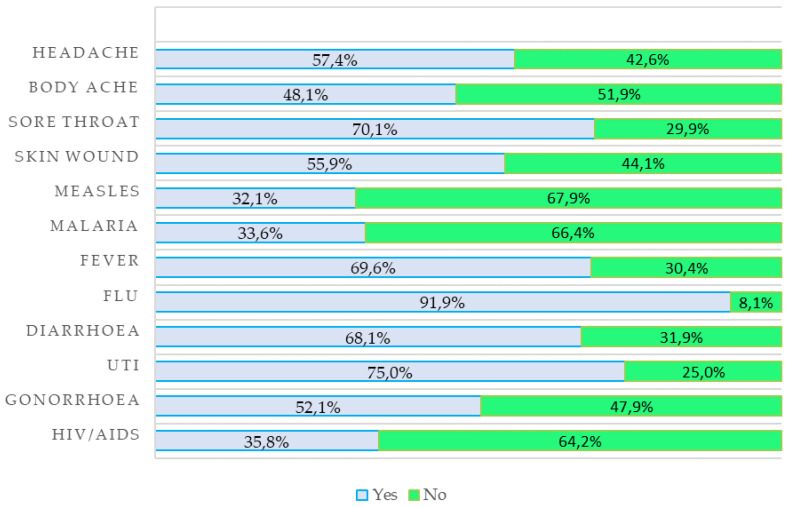
Knowledge of conditions that can be treated with antibiotics.

Figure 1 presents the responses to questions about conditions that can be treated with antibiotics. More than half of the respondents (57.4%) believed that headaches can be treated with antibiotics, and 48.1% believed that antibiotics can relieve body aches. Notably, 67.9% of respondents were not aware that measles is a condition that can be managed with antibiotics. Additionally, 69.9% replied that they would use antibiotics in case of fever, and a high percentage of 91.9% agreed with the statement that antibiotics are required for the common flu.

## 3. Discussion

This survey aimed to evaluate the level of awareness regarding antibiotic use and resistance among the adult population of Kazakhstan. Half of the respondents (50.4%) received antibiotic therapy within the last 12 months, of which 40.1% had no prescription and 40.4% did not receive advice from a medical professional on how to take them. Nearly two-thirds of respondents (65.3%) had never heard of antibiotic resistance, and 57.2% believed that it is acceptable to request the same antibiotic if it had helped to treat the same symptoms previously. In general, knowledge about antibiotic use for specific health conditions was found to be low in 82.1% of the study participants.

Our study findings are consistent with reports from other Eastern European countries. A study by Zajmi et al. in Kosovo showed that more than half of the respondents (58.7%) used antibiotics in the past year, and a quarter (25.0%) took them without a physician’s prescription. Notably, 42.5% of respondents believed that viral infections could be effectively treated with antibiotics [7]. In Georgia, over half (55%) of adults received antibiotics without consulting a medical professional, and 62% bought antibiotics without a prescription [8]. A study in Lithuania revealed that 61.1% of respondents had poor knowledge of antibiotics, and 26.0% believed that antibiotics are effective against viral infections [9]. In Serbia, 58.4% of respondents considered antibiotics to be effective against the common cold [10]. In Romania, more than half (61.45%) of the general public received antibiotics at least once in the past year, and only 57.43% reported consulting a physician before taking them [11]. However, a study from Poland reported lower rates of antibiotic consumption in the previous year (38.0%). Unlike the findings of other Eastern European studies, the majority of antibiotics (90%) were prescribed by a doctor [12].

To mitigate the problem of antibiotic resistance, the sale of antibiotics without a prescription should be prohibited, and self-medication should be discouraged. Nevertheless, it is a common practice in many developing countries to sell antibiotics upon a patient’s request. A qualitative study from India confirmed that pharmacists readily admitted to selling antibiotics over the counter and were generally unaware of the issue of antibiotic resistance [13]. In Damascus, Syria, 87% of pharmacy workers easily agreed to sell antibiotics without a prescription, and 97% sold antibiotics if a patient insisted [14]. Another study from the Middle East reported high rates of over-the-counter sales of antibiotics (63.6%) in Saudi Arabia [15]. Both online and community pharmacies sell antibiotics without a valid prescription, as a study from China showed. However, community pharmacies were more likely to sell antibiotics over the counter and provide no necessary information to patients [16].

Poor or suboptimal knowledge of appropriate antibiotic use is another cause for global concern, as it significantly contributes to antibiotic resistance. This includes awareness of the spectrum of diseases that can be treated with antibiotics, the duration of therapy, and understanding when it is appropriate to stop taking them. Akhund et al. conducted an online survey in Pakistan and found that out of 1132 participants, 837 (73.9%) believed that it is possible to stop the course of antibiotics whenever they feel better, and 505 (44.6%) were convinced that frequent and unnecessary use of antibiotics reduces their effectiveness. Notably, 157 (13.9%) of the participants did not adhere to the duration of treatment recommended by a doctor. As many as 467 (41.3%) of the respondents reused antibiotics left over from a previous prescription when experiencing similar symptoms [17]. In our study, 33.2% of respondents stopped their intake of antibiotics when they felt better. Inadequate knowledge concerning the time to stop antibiotic therapy was also reported by other researchers [14,15,16].

Self-medication with antibiotics is becoming widespread, and many people are convinced that they can use the same antibiotic for a condition with similar symptoms. In this study, more than half of the participants (57.2%) thought it is acceptable to buy the same antibiotic or request it from a doctor if it helped them to get better when the same symptoms were present before. In a study from Romania, 10.34% of respondents took antibiotics following recommendations of a family member or friend, and 22.9% used the same antibiotic prescribed by a doctor at the last visit [11]. In general, the population of Eastern Europe has higher rates of self-medication with antibiotics as compared to the population of Western Europe. This could be attributed to cultural differences and law enforcement efforts to prohibit the over-the-counter sale of prescription drugs [18].

A striking finding of this study is that a high proportion of the population (91.9%) is ready to take antibiotics for a common cold. This is probably best explained by the incorrect identification of bacteria as the most common cause of upper respiratory tract infections. The same misconception is shared by the populations of other countries, although to a lesser extent. In Germany, 10.5% of patients asked for antibiotics to treat common colds [19], and 14% of patients in Denmark requested a prescription of antibiotics for upper respiratory tract infections [20]. This proportion is declining in Romania, where as many as 51% of respondents considered antibiotics effective against flu in 2009 [21], compared to 39% in 2016 [22]. Indeed, our findings are comparable to data from Myanmar, where 72.6% of respondents believed that antibiotics can eliminate viruses and 73.5% considered antibiotics effective against the flu. Interestingly, these beliefs were more prevalent among younger individuals and those residing in urban areas [23].

Another commonly shared misbelief is that antibiotics can be used to treat non-bloody diarrhea. According to a systematic review by Carter et al., low- and middle-income countries tend to over-rely on antibiotics in the treatment of childhood diarrhea as they are prescribed in 10–77% of cases [24]. Another meta-analysis by Edessa et al. showed that the rate of non-prescribed antibiotics in pediatric practice at the community level in low- and middle-income countries constitute 45% [25].

There is a need for awareness-raising campaigns to improve knowledge about antibiotic use and resistance in the population of Kazakhstan. This could be done via social media, and the importance of seeking professional advice before initiating antibacterial therapy has to be emphasized. The country’s medical community, in particular the primary healthcare sector, should adopt the best health education strategies, and policymakers have to reinforce their efforts to control the over-the-counter sale of prescription medications. Currently, there is a lack of recognition of antibiotic misuse in Kazakhstan, and therefore it is important to sensitize all stakeholders. The country’s stakeholders can benefit from the strategies proposed by World Antimicrobial Awareness Week, which was designed to enable communication with a focus on effective approaches to prevent and mitigate antimicrobial resistance [26].

The current study has several limitations primarily stemming from its cross-sectional design. One of the main limitations is the inability to establish causal relationships between knowledge and practice of antibiotic use and individual participant characteristics. Originally, our intention was to sample healthy individuals visiting outpatient facilities for routine check-ups or accompanying their diseased relatives. However, we quickly realized that few adults attend healthcare facilities for preventive purposes. As a result, the majority of participants in our study were accompanying persons, leading to observed shifts in gender and age. Therefore, this sample is not representative of the entire population of Kazakhstan and should be considered as a pilot study. Despite these limitations, our study has many strengths. It is based on a reasonably large sample of the adult population enrolled from a typical region of the country. Furthermore, it is the first study to investigate awareness about antibiotic use and resistance among the population of Kazakhstan and can serve as a benchmark for future research.

## 4. Materials and Methods

### 4.1. Study Design and Procedures

A cross-sectional study was conducted between October 2021 and February 2022, using a random sample of adult residents without medical education in the East Kazakhstan region. This region was selected as a representative area of the country, based on socio-economic indicators, and had an estimated population of 1,349,400 in 2022 [27]. The sample size was calculated using the Sample XS calculator from Brixton Health [28], for a population size of 13 million (the adult population of Kazakhstan), 80% power, an estimated prevalence of 20%, and a design effect of 1.0. The resulting sample size was 683 individuals, but we enrolled 750 participants to allow for potential dropouts. Ultimately, 727 individuals agreed to participate, resulting in a response rate of 96.9%.

We recruited healthy individuals attending regional outpatient facilities for routine check-ups or as accompanying persons, using a systematic random sampling method. Out of 35 outpatient polyclinic organizations located on the territory of the East Kazakhstan region, 15 were sampled to represent two cities—Ust-Kamenogorsk and Semey (5 facilities from each city) and rural territories. We sampled 5 rural outpatient facilities located in the region’s north-west (Beskaragay district), north-east (Altai district), center (Zharma district), south-west (Ayagoz district), and south-east (Tarbagatay district). A maximum of 15–20 individuals were recruited from each clinic per day. The full WHO questionnaire was administered through face-to-face interviews. Age below 18 years, presence of medical education, and unwillingness to participate in the study served as the exclusion criteria.

### 4.2. The Tool and Data Collection Techniques

This survey followed the methodology described in the WHO report “Antibiotic Resistance: Multi-country public awareness survey” [29]. The questionnaire consisted of four sections. The first section included questions related to the social and demographic characteristics of respondents, while the second and third sections focused on the knowledge and use of antibiotics. Knowledge about antibiotic resistance was assessed in the fourth section. The questionnaire was administered in the Kazakh and Russian languages and it took approximately 10–15 min to complete it. The study aim was clearly explained to the participants before the data collection and informed consent was obtained. To keep confidentiality, identity information was not collected. All data were encrypted and stored electronically in a secure location and a password was solely available to the principal investigator (N.I.) to ensure the privacy of study participants.

### 4.3. Statistical Analysis

The study data were analyzed using the Statistical Package for Social Sciences (SPSS) version 20. As a first step of statistical analysis, the type of data distribution was evaluated for continuous variables by the Kolmogorov-Smirnov test. As the distribution of data proved to be different from normal, the continuous variables were presented as the median with interquartile ranges and non-parametric tests were used to compare differences between the groups. Categorical variables were presented as the frequency with percentage and Pearson’s chi-square test was utilized for between-group comparisons. A *p*-value of 0.05 and below was considered statistically significant.

To facilitate educational level comparisons, we classified educational attainments into two categories: pre-tertiary (including no schooling, completed school, some college credit, and non-degree technical/vocational training) and tertiary (comprising bachelor’s, master’s, and doctoral (Ph.D.) degrees). To elucidate the differences between groups based on place of residence, we compared individuals living in urban and suburban areas with individuals living in rural areas. To compare responses from different income groups, we divided all respondents into two groups based on their monthly income levels: a lower income group (monthly income ≤150,000 Tenge) and a higher income group (monthly income exceeding 150,000 Tenge). At the time of the survey in 2021, 150,000 Tenge was approximately equivalent to 350 US dollars [30].

### 4.4. Ethical Considerations

This study has been approved by the Ethics Committee of Semey Medical University with registration code 2, dated 28 October 2020.

## 5. Conclusions

This study sheds light on the level of awareness and behavior regarding antibiotic use and resistance in Kazakhstan, with a focus on gender, age, education, place of residence, and income level. The findings reveal significant differences in the duration since respondents last received antibiotics, prescription patterns, beliefs about antibiotic use, and seeking advice from healthcare professionals between males and females. Moreover, age, education, and income level were associated with differences in antibiotic use and awareness. Our study highlights the importance of increasing awareness and promoting the appropriate use of antibiotics among different population groups in Kazakhstan. Further research and interventions are needed to address the identified differences and improve antibiotic use practices in the country.

## Figures and Tables

**Table 1:** General characteristics of the study participants.

General Characteristics (n = 727)		n	%
Sex	Males	185	25.4
	Females	542	74.6
Age	18–24	438	60.2
	25–34	114	15.7
	35–44	72	9.9
	45–54	36	5.0
	55–64	48	6.6
	65+	19	2.6
Location	Urban	390	53.6
	Suburban	200	27.5
	Rural	137	18.8
Education	No schooling completed	31	4.3
	Only school completed	151	20.8
	Some college credit, no degree	41	5.6
	Technical/Vocational training	101	13.9
	Bachelor’s degree	343	47.2
	Master’s degree	53	7.3
	Doctorate (Ph.D.) degree	7	1.0
Household composition	Single adult only	144	19.8
	Single adult and at least 1 child under 16	15	2.1
	Married adults only	22	3.0
	Married and at least 1 child under 16	132	18.2
	Multiple adults aged 16+ only	120	16.5
	Multiple adults aged 16+ only and at least 1 child under 16	294	40.4
Household income, median, 25–75 percentiles		150,000	(100,000–300,000)

**Table 2:** Awareness about antibiotic use and resistance by sex.

Questions		Male		Female		*p*-Value
		n	%	n	%	
When do you think you should stop taking the antibiotics? (n = 620)	When you feel better	67	45.0	139	29.5	<0.001
	When you’ve taken all of the antibiotics as directed	82	55.0	332	70.5	
Do you think it is good to use the same antibiotic if a friend or family member used to treat same symptoms or disease before? (n = 548)	True	47	34.3	69	16.8	<0.001
	False	90	65.7	342	83.2	
Do you think it is good to ask/request the same antibiotic if it helped to treat the same symptoms/disease previously? (n = 516)	True	75	57.7	220	57.0	0.889
	False	55	42.3	166	43.0	
Have you heard term “Antibiotic resistance”? (n = 727)	Yes	67	36.2	185	34.1	0.607
	No	118	63.8	357	65.9	
When did you last take antibiotics? (571)	In the last month	49	33.1	109	25.8	0.014
	In the last 6 months	25	16.9	122	28.8	
	In the last year	12	8.1	50	11.8	
	More than a year ago	28	18.9	58	13.7	
	Never	34	23.0	84	19.9	
Did you have a prescription for this antibiotic? (n = 636) Did you get advice from a doctor, nurse or pharmacist on how to take them? (n = 628)	Yes	78	47.9	303	64.1	<0.001
	No	85	52.1	170	35.9	
	Yes	72	46.8	302	63.7	<0.001
	No	82	53.2	172	36.3	
Where did you get the antibiotics? (n = 650)	Medical store or pharmacy	156	95.7	479	98.4	0.009
	The internet	3	1.8	2	0.4	
	Friend or family member	1	0.6	6	1.2	
	I had them saved up from a previous time	3	1.8	0	0.0	

**Table 3:** Awareness about antibiotic use and resistance by age.

Questions		18–24 Years of Age		25 Years and Older		*p*-Value
		n	%	n	%	
When do you think you should stop taking the antibiotics? (n = 620)	When you feel better	123	34.0	83	32.2	0.638
	When you’ve taken all of the antibiotics as directed	239	66.0	175	67.8	
Do you think it is good to use the same antibiotic if a friend or family member used to treat same symptoms or disease before? (n = 548)	True	61	18.1	55	26.1	0.026
	False	276	81.9	156	73.9	
Do you think it is good to ask/request the same antibiotic if it helped to treat the same symptoms/disease previously? (n = 516)	True	165	53.9	130	61.9	0.072
	False	141	46.1	80	38.1	
Have you heard term “Antibiotic resistance”? (n = 727)	Yes	137	31.3	115	39.8	0.018
	No	301	68.7	174	60.2	
When did you last take antibiotics? (571)	In the last month	92	28.3	66	26.8	<0.001
	In the last 6 months	68	20.9	79	32.2	
	In the last year	29	8.9	33	13.4	
	More than a year ago	41	12.6	45	18.3	
	Never	95	29.3	23	9.3	
Did you have a prescription for this antibiotic? (n = 636) Did you get advice from a doctor, nurse or pharmacist on how to take them? (n = 628)	Yes	222	58.4	159	62.1	0.352
	No	158	41.6	97	37.9	
	Yes	213	55.6	161	65.7	0.012
	No	170	44.4	84	34.3	
Where did you get the antibiotics? (n = 650)	Medical store or pharmacy	378	97.7	257	97.8	0.272
	The internet	2	0.5	3	1.1	
	Friend or family member	4	1.0	3	1.1	
	I had them saved up from a previous time	3	0.8	0	0.0	

**Table 4:** Awareness about antibiotic use and resistance by education level.

Questions		Pre-Tertiary Education		Tertiary Education		*p*-Value
		n	%	n	%	
When do you think you should stop taking the antibiotics? (n = 620)	When you feel better	99	35.6	107	31.3	0.256
	When you’ve taken all of the antibiotics as directed	179	64.4	235	68.7	
Do you think it is good to use the same antibiotic if a friend or family member used to treat same symptoms or disease before? (n = 548)	True	57	24.8	59	18.6	0.078
	False	173	75.2	259	81.4	
Do you think it is good to ask/request the same antibiotic if it helped to treat the same symptoms/disease previously? (n = 516)	True	123	54.4	172	59.3	0.266
	False	103	45.6	118	40.7	
Have you heard term “Antibiotic resistance”? (n = 727)	Yes	101	31.2	151	37.5	0.076
	No	223	68.8	252	62.5	
When did you last take antibiotics? (571)	In the last month	81	30.3	77	25.3	0.039
	In the last 6 months	59	22.1	88	28.9	
	In the last year	22	8.2	40	13.2	
	More than a year ago	48	18.0	38	12.5	
	Never	57	21.3	61	20.1	
Did you have a prescription for this antibiotic? (n = 636) Did you get advice from a doctor, nurse or pharmacist on how to take them? (n = 628)	Yes	149	53.8	232	64.6	0.006
	No	128	46.2	127	32.4	
	Yes	156	58.6	218	60.2	0.691
	No	110	41.4	144	39.8	
Where did you get the antibiotics? (n = 650)	Medical store or pharmacy	274	96.5	361	98.6	0.172
	The internet	3	1.1	2	0.5	
	Friend or family member	4	1.4	3	0.8	
	I had them saved up from a previous time	3	1.1	0	0.0	

**Table 5:** Awareness about antibiotic use and resistance by the place of residence.

Questions		Urban and Suburban Residents		Rural Residents		*p*-Value
		n	%	n	%	
When do you think you should stop taking the antibiotics? (n = 620)	When you feel better	164	32.4	42	36.8	0.364
	When you’ve taken all of the antibiotics as directed	342	67.6	72	63.2	
Do you think it is good to use the same antibiotic if a friend or family member used to treat same symptoms or disease before? (n = 548)	True	99	22.4	17	16.0	0.150
	False	343	77.6	89	84.0	
Do you think it is good to ask/request the same antibiotic if it helped to treat the same symptoms/disease previously? (n = 516)	True	239	57.0	56	57.7	0.901
	False	180	43.0	41	42.3	
Have you heard term “Antibiotic resistance”? (n = 727)	Yes	208	35.3	44	32.1	0.487
	No	382	64.7	93	67.9	
When did you last take antibiotics? (571)	In the last month	120	25.6	38	36.9	0.010
	In the last 6 months	121	25.9	26	25.2	
	In the last year	58	12.4	4	3.9	
	More than a year ago	66	14.1	20	19.4	
	Never	103	22.0	15	14.6	
Did you have a prescription for this antibiotic? (n = 636) Did you get advice from a doctor, nurse or pharmacist on how to take them? (n = 628)	Yes	309	58.7	72	65.5	0.192
	No	217	41.3	38	34.5	
	Yes	309	59.8	65	58.6	0.814
	No	208	40.2	46	41.4	
Where did you get the antibiotics? (n = 650)	Medical store or pharmacy	527	98.0	108	96.4	0.273
	The internet	4	0.7	1	0.9	
	Friend or family member	4	0.7	3	2.7	
	I had them saved up from a previous time	3	0.6	0	0.0	

**Table 6:** Awareness about antibiotic use and resistance by the level of income.

Questions		Lower Income		Higher Income		*p*-Value
		n	%	n	%	
When do you think you should stop taking the antibiotics? (n = 620)	When you feel better	117	34.7	89	31.4	0.389
	When you’ve taken all of the antibiotics as directed	220	65.3	194	68.6	
Do you think it is good to use the same antibiotic if a friend or family member used to treat same symptoms or disease before? (n = 548)	True	61	20.8	55	21.6	0.830
	False	232	79.2	200	78.4	
Do you think it is good to ask/request the same antibiotic if it helped to treat the same symptoms/disease previously? (n = 516)	True	156	53.1	139	62.6	0.030
	False	138	46.9	83	37.4	
Have you heard term “Antibiotic resistance”? (n = 727)	Yes	126	30.4	126	40.3	0.006
	No	288	69.6	187	59.7	
When did you last take antibiotics? (571)	In the last month	90	28.8	68	26.4	0.004
	In the last 6 months	63	20.1	84	32.6	
	In the last year	34	10.9	28	10.9	
	More than a year ago	59	18.8	27	10.5	
	Never	67	21.4	51	19.8	
Did you have a prescription for this antibiotic? (n = 636) Did you get advice from a doctor, nurse or pharmacist on how to take them? (n = 628)	Yes	203	59.0	178	61.0	0.618
	No	141	41.0	114	39.0	
	Yes	200	58.8	174	60.4	0.685
	No	140	41.2	114	39.6	
Where did you get the antibiotics? (n = 650)	Medical store or pharmacy	341	97.4	294	98.0	0.092
	The internet	3	0.9	2	0.7	
	Friend or family member	6	1.7	1	0.3	
	I had them saved up from a previous time	0	0.0	3	1.0	

## Data Availability

The data presented in this study are available on a reasonable request from the corresponding author.
